# Carbapenem is not always the best choice in the treatment of septic shock

**DOI:** 10.1186/s40001-023-01341-x

**Published:** 2023-09-25

**Authors:** Lu Wang, Xudong Ma, Yujie Chen, Sifa Gao, Huaiwu He, Longxiang Su, Yanhong Guo, Guangliang Shan, Yaoda Hu, Xiang Zhou, Dawei Liu, China National Critical Care Quality Control Centre Group (China-NCCQC), Xue Wang, Xiangdong Guan, Yan Kang, Bin Xiong, Bingyu Qin, Kejian Qian, Chunting Wang, Mingyan Zhao, Xiaochun Ma, Xiangyou Yu, Jiandong Lin, Aijun Pan, Haibo Qiu, Feng Shen, Shusheng Li, Yuhang Ai, Xiaohong Xie, Jing Yan, Weidong Wu, Meili Duan, Linjun Wan, Xiaojun Yang, Jian Liu, Hang Xu, Dongpo Jiang, Lei Xu, Zhuang Chen, Guoying Lin, Zhengping Yang, Zhenjie Hu

**Affiliations:** 1 Department of Critical Care Medicine, State Key Laboratory of Complex Severe and Rare Diseases, Peking Union Medical College Hospital, Peking Union Medical College and Chinese Academy of Medical Sciences, Beijing, 100730 China; 2 Department of Medical Administration, National Health Commission of the People’s Republic of China, Beijing, 100044 China; 3 Department of Epidemiology and Biostatistics, Institute of Basic Medicine Sciences, Chinese Academy of Medical Sciences (CAMS) & School of Basic Medicine, Peking Union Medical College, Beijing, 100730 China; 4 Information Center Department/Department of Information Management, Peking Union Medical College Hospital, Peking Union Medical College and Chinese Academy of Medical Sciences, Beijing, 100730 China

**Keywords:** Septic shock, Carbapenem, Fermenting bacteria, Mortality, Hospital management

## Abstract

**Background:**

Septic shock is a global public health burden. In addition to the improvement of the level of individual care, the improvement of the overall hospital quality control management is also an essential key aspect of the Surviving Sepsis Campaign (SSC). Using of antibiotics is a cornerstone in the treatment of septic shock, so we conducted this study to investigate the influence of antibiotics and pathogenic bacteria on the mortality of septic shock at the level of overall hospital in China.

**Methods:**

This was an observational database study in 2021 enrolled the data of 787 hospitals from 31 provinces/municipalities/autonomous regions of Mainland China collected in a survey from January 1, 2021 to December 31, 2021.

**Results:**

The proportion of ICU patients with septic shock was 3.55%, while the patient mortality of septic shock was 23.08%. While carbapenem was the most preferred antibiotic medication used in 459 of the 782 hospitals, the preference for carbapenem did not show significant effect on the patient mortality in the treatment of septic shock (*p*-value 0.59). Compared with patients with fermenting bacteria as the most common pathogenic bacteria causing septic shock, patients with non-fermenting bacteria had a higher mortality (*p*-value 0.01).

**Conclusions:**

Whether using carbapenem as the preferred antibiotic or not, did not show effect on the patient mortality of septic shock. Compared with patients with fermenting bacteria as the most common pathogenic bacteria, patients of septic shock with non-fermenting bacteria had a higher mortality.

## Background

Sepsis is an organ dysfunction caused by the host's dysfunctional response to infection, becomes ultimately a life-threatening disease [1]. Septic shock accounted for 10% of patients admitted to intensive care units [2]. Septic shock is the most severe form of sepsis, with an estimated incidence of 20 per 100,000 population [3]. Intensive care unit (ICU), hospitalization, and one-year mortality for septic shock are 37–47%, 39–56%, and 60%, respectively [3]. In addition to the improvement at the level of individual care, the improvement at the level of overall hospital quality control management is also a key aspect [4, 5]. A pre-established multistep bundles intervention can improve clinical management and outcomes of patients with Gram-negative bloodstream infection [6]. In previous studies, our research group discussed the association of the annual hospital septic shock case volume and the hospital patient mortality [7], as well as the influence factors affecting sepsis 1-h, 3-h and 6-h SSC bundle compliance [8–10]. Early resuscitation, antibiotics, and source control are the three cornerstones of the treatment of septic shock. Because patients with septic shock are often critically ill, the concept of hammer punching is still widely popular, and the use of carbapenem is widespread. Therefore, due to the widespread use of carbapenem, the problems of antibiotic resistance and the epidemic of non-fermenting bacteria are also becoming increasingly prominent. Twenty-eight percent of ICU patients tested positive for carriage of *Klebsiella pneumoniae* immediately upon admission, 54% of which were carbapenem-resistant [11]. Carbapenem-resistant *A. baumannii* strains were prevalent in 71.4% of the ICUs in China [12]. We conducted this study to investigate the influence of antibiotics and pathogenic bacteria on the mortality of septic shock at the level of overall hospital in China.

## Methods

### Design

This was an observational database study from 2021 based on the data source from the National Clinical Improvement System (https://ncisdc.medidata.cn/login.jsp), collected by the China-National Critical Care Quality Control Centre (China-NCCQC). The Quality Improvement of Critical Care (QICC) Program, led by China-NCCQC, was initiated in 2015, while this study is part of the above program.

### Study population and settings

A total of 787 hospitals in China were enrolled in this survey study. The ICUs in these hospitals admitted a total of 674,485 patients, including 56,591 patients with septic shock. The enrolled hospitals from 31 provinces/municipalities/autonomous regions of Mainland China participated voluntarily in the study and were selected by referring to the following criteria: (1) number of patients admitted to the ICU ≥ 100/year. (2) Number of patients with septic shock admitted to the ICU ≥ 10/year. (3) The ICU needs to have the ability to diagnose infections caused by non-fermenting bacteria and fermenting bacteria. Severe patients are defined as patients with Acute Physiology and Chronic Health Evaluation (APACHE) II score ≥ 15.

Hospitals providing treatment for patients with septic shock in China are mainly tertiary and secondary hospitals. Tertiary hospitals usually serve as medical hubs providing care on the supra-regional level, while secondary hospitals are responsible for providing comprehensive health services on the regional basis [13], wherefore we investigated the two types of hospitals separately.

### Variables and measurements

Whether using carbapenem as the preferred antibiotic medication in the treatment of septic shock and whether detecting non-fermenting bacteria as the most common pathogenic bacteria causing septic shock were measured as variables. The preferred antibiotic medication in the treatment of septic shock means the most frequent antibacterial drugs used among the patients admitted to the ICU this year with septic shock. The most common pathogenic bacteria causing septic shock means the infectious pathogenic bacteria ranked first among the patients admitted to the ICU this year with septic shock.

### Data collection

The data collection was completed between January 1, 2021 and December 31, 2021. Informed consent was obtained from every study participating hospital’s ethic committees. The collected data were transferred into a data analysis system by an independent research coordinator.

### Data analysis

Based on the data obtained from this survey, we first try to find out the effects of whether using carbapenem as the preferred antibiotic medication in the treatment of septic shock and whether detecting non-fermenting bacteria as the most common pathogenic bacteria causing septic shock on the patient mortality of septic shock. Then we analyzed the above effects in terms of tertiary and secondary hospitals.

### Ethical considerations

The current study was reported in accordance with the Strengthening the Reporting of Observational Studies in Epidemiology Guidelines. The study was conducted in accordance with the Declaration of Helsinki (as revised in 2013). The trial protocol was approved by the Central Institutional Review Board at Peking Union Medical College Hospital (NO. SK1828), while individual consent for this analysis was waived. There were no identifying or protected health information included in the analyzed dataset. In addition, all participating hospitals received the approval by their local research ethics boards, with written consent obtained on the hospital-level from the hospital medical directors. The authors are accountable for all aspects of the work in ensuring that questions related to the accuracy or integrity of any part of the work are appropriately investigated and resolved.

### Statistical analysis

All statistical analyses were performed in SAS 9.4 (SAS Institute Inc., Cary, NC, USA). Information of septic shock patients were expressed as mean ± standard deviation. The patient mortality caused by septic shock was expressed as the mean ± standard deviation and media (P25, P75). The unpaired t-test was used to test the basic condition of septic shock patients. The binary logistic regression model and binary statistical analysis were used to investigate the effects of whether using carbapenem as the preferred antibiotic medication in the treatment of septic shock and whether detecting non-fermenting bacteria as the most common pathogenic bacteria causing septic shock on the patient mortality caused by septic shock, and to conduct subsequent subgroup analysis. All statistical tests were two-tailed, while *p* < 0.05 was considered to be statistically significant.

## Results

**Fig. 1:**
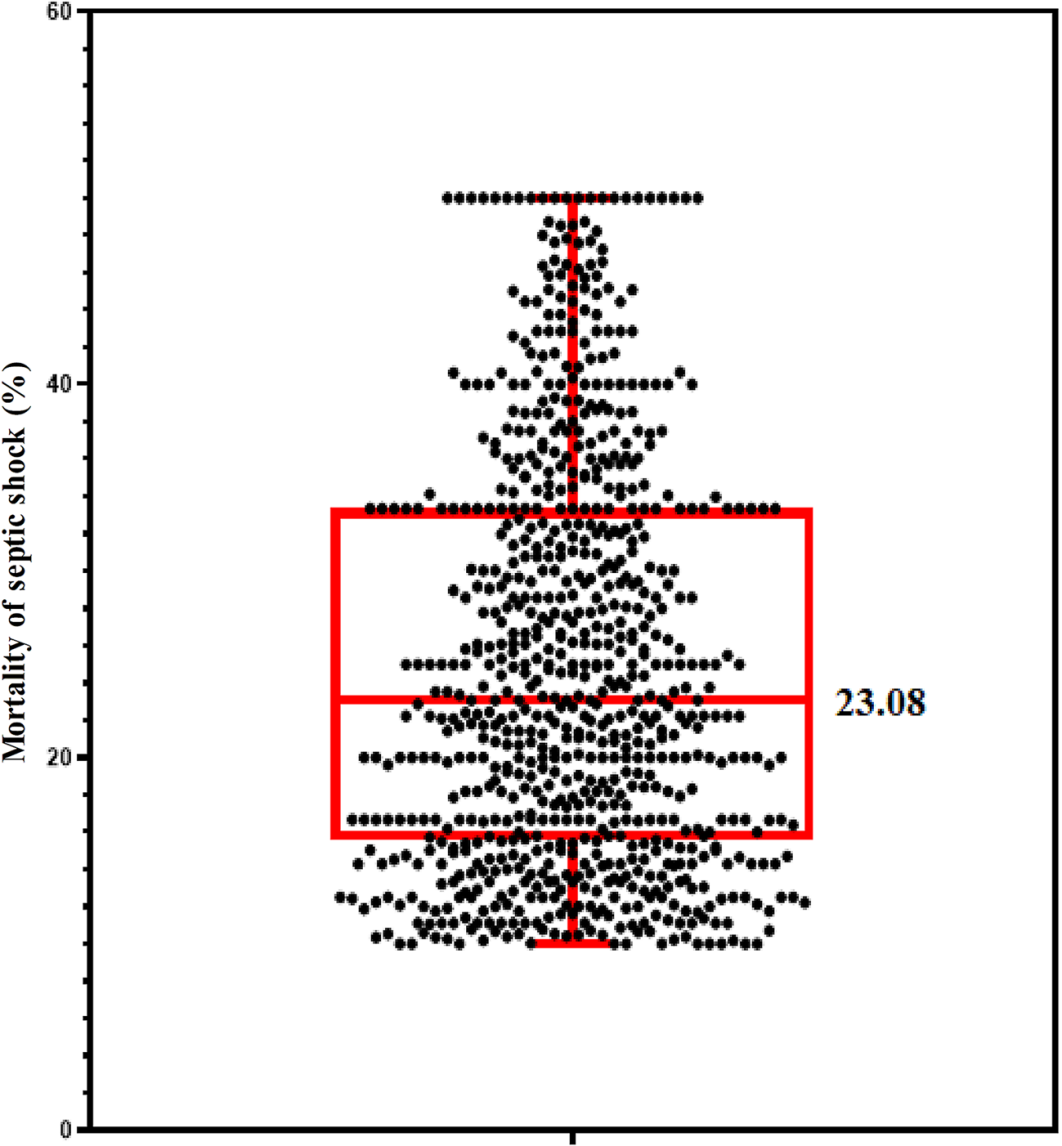
Patient mortality of septic shock

In the treatment of septic shock, 459 hospitals used carbapenem as the preferred antibiotic treatment, while only 323 hospitals used non-carbapenem as the preferred antibiotic treatment, except 5 hospitals without relevant data. In terms of detecting the most common pathogenic bacteria causing septic shock, 131 hospitals detected non-fermenting bacteria and 602 hospitals detected fermenting bacteria. Hereby, 49 hospitals detected other infection-based bacteria groups, while 5 hospitals were without relevant data. The patient mortality caused by septic shock was 25.05 ± 10.90, 23.08 (15.56, 33.33) (Fig. 1).

There were no statistically significant differences in sex, age, or proportion of severe patients in the group between patients whether using carbapenem as the preferred antibiotic medication in the treatment of septic shock (Table 1). There were no statistically significant differences in sex, or age between patients whether detecting non-fermenting bacteria as the most common pathogenic bacteria causing septic shock (Table 2). The proportion of severe patients in patients detecting non-fermenting bacteria as the most common pathogenic bacteria causing septic shock was lower than it in patients detecting fermenting bacteria as the most common pathogenic bacteria causing septic shock (*p*-value 0.03) (Table 2).

**Table 1:** Information of septic shock patient with different antibiotics

Proportion of patients (%)	With carbapenem	Without carbapenem	*P*
Male	58.97 ± 0.56	60.49 ± 1.16	0.25
Age			
< 18	1.92 ± 0.42	0.77 ± 0.40	0.05
[18, 30]	2.30 ± 0.15	2.32 ± 0.33	0.95
[31, 40]	4.61 ± 0.24	5.20 ± 0.62	0.32
[41, 50]	9.08 ± 0.34	8.34 ± 0.62	0.34
[51, 60]	16.37 ± 0.48	16.39 ± 1.09	0.99
[61, 70]	23.62 ± 0.51	22.95 ± 0.96	0.57
[71, 80]	23.97 ± 0.58	24.49 ± 1.20	0.70
> 80	15.97 ± 0.57	17.26 ± 1.38	0.35
Severe patients	59.48 ± 1.05	62.69 ± 2.08	0.19

Severe patients are defined as patients with Acute Physiology and Chronic Health Evaluation (APACHE) II score ≥ 15

*With carbapenem* using carbapenem as the preferred antibiotic medication, *Without carbapenem* not using carbapenem as the preferred antibiotic medication

**Table 2:** Information of septic shock patient with different pathogenic bacteria

Proportion of patients (%)	Non-fermenting	Fermenting	*P*
Male	58.68 ± 0.83	59.63 ± 0.64	0.36
Age			
< 18	1.19 ± 0.48	2.08 ± 0.50	0.20
[18, 30]	2.10 ± 0.24	2.44 ± 0.17	0.24
[31, 40]	4.87 ± 0.40	4.60 ± 0.27	0.57
[41, 50]	9.68 ± 0.53	8.44 ± 0.34	0.05
[51, 60]	16.96 ± 0.80	15.97 ± 0.50	0.30
[61, 70]	23.63 ± 0.77	23.41 ± 0.56	0.81
[71, 80]	23.3 ± 0.82	24.6 ± 0.67	0.22
> 80	15.94 ± 0.88	16.38 ± 0.66	0.68
Severe patients	57.57 ± 1.53	61.78 ± 1.18	0.03

Severe patients are defined as patients with Acute Physiology and Chronic Health Evaluation (APACHE) II score ≥ 15

*Non-fermenting* detecting non-fermenting bacteria as the most common pathogenic bacteria causing septic shock, *Fermenting* detecting fermenting bacteria as the most common pathogenic bacteria causing septic shock

**Fig. 2:**
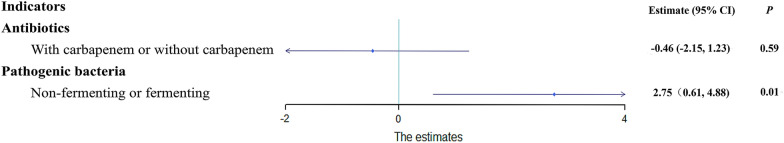
Effects of antibiotics and pathogenic bacteria on the patient mortality of septic shock. *With carbapenem* using carbapenem as the preferred antibiotic medication, *without carbapenem* not using carbapenem as the preferred antibiotic medication. *Non-fermenting* detecting non-fermenting bacteria as the most common pathogenic bacteria causing septic shock, *fermenting* detecting fermenting bacteria as the most common pathogenic bacteria causing septic shock

**Fig. 3:**
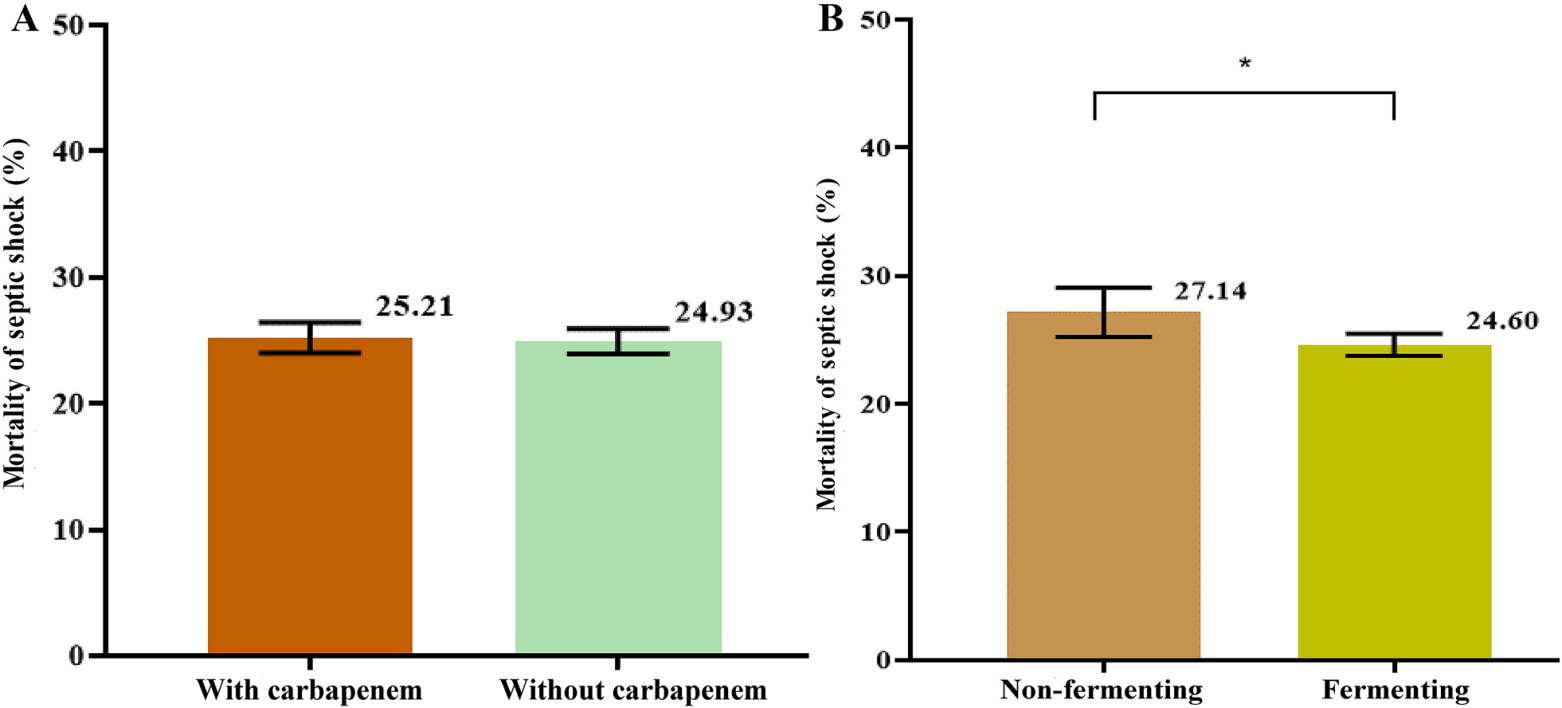
Effects of antibiotics and pathogenic bacteria on the patient mortality of septic shock. *With carbapenem* using carbapenem as the preferred antibiotic medication, *without carbapenem* not using carbapenem as the preferred antibiotic medication. *Non-fermenting* detecting non-fermenting bacteria as the most common pathogenic bacteria causing septic shock, *fermenting* detecting fermenting bacteria as the most common pathogenic bacteria causing septic shock.* *p* < 0.05, compared with the Non-fermenting group.

In the treatment of septic shock, whether to use carbapenem as preferred antibiotic had no effect on the patient mortality of septic shock (*p*-value 0.59) (Fig. 2), and the result was consistent in binary statistical analysis (*p*-value 0.12) (Fig. 3) and classification analysis by tertiary hospitals (*p*-value 0.94) and secondary hospitals (*p*-value 0.57) (Table 3). Compared with patients with fermenting bacteria as the most common pathogenic bacteria causing septic shock, patients with non-fermenting bacteria had a higher mortality (*p*-value 0.01) (Fig. 2), and the results of binary statistical analysis (*p*-value 0.03) (Fig. 3) and classification analysis by secondary hospitals were consistent (*p*-value 0.04) (Table 3). Only the *p*-value of tertiary hospitals was 0.06 (Table 3).

**Table 3:** Effects of antibiotics and pathogenic bacteria on the patient mortality of septic shock among hospitals of different grades

Indicators	Tertiary hospital (*n* = 498)		Secondary hospital (*n* = 289)	
	Estimate (95%CI)	*P*	Estimate (95%CI)	*P*
Antibiotics				
With carbapenem or without carbapenem	− 0.08 (− 2.24, 2.07)	0.94	− 0.83 (− 3.71, 2.04)	0.57
Pathogenic bacteria				
Non-fermenting or fermenting	2.39 (− 0.14, 4.91)	0.06	4.19 (0.09, 8.28)	0.04

*With carbapenem* using carbapenem as the preferred antibiotic medication, *Without carbapenem* not using carbapenem as the preferred antibiotic medication, *Non-fermenting* detecting non-fermenting bacteria as the most common pathogenic bacteria causing septic shock, *Fermenting* detecting fermenting bacteria as the most common pathogenic bacteria causing septic shock

## Discussion

Septic shock refers to the development of fluid-refractory hypotension requiring vasopressors, and is associated with tissue hypoperfusion (lactate > 2 mmol/L) in a patient with sepsis [1]. Septic shock requires prompt identification, appropriate antibiotic treatment, intensive hemodynamic support, and control of infection focus [14]. As a major challenge in the field of global public health, improvement of overall quality control management in hospitals is also an important aspect of the SSC [15–17]. Therefore, we conducted this study to investigate the influence of antibiotics and pathogenic bacteria on septic shock from the perspective of overall hospital.

Although the problem of drug resistance due to abuse has been repeatedly emphasized, carbapenem as the preferred antibiotic medication still ranked first in the antibiotic treatment regime of septic shock in the present study. Carbapenem was used as the preferred antibiotic in 459 of the 782 hospitals, which marks the abuse of carbapenem antibiotics has become an increasingly prominent public health problem. In our research, whether to use carbapenem as preferred antibiotic or not, did not show effect on the mortality of septic shock. One of the potential explanations for this phenomenon could be that the majority of the infections were caused by bacteria strains, which were not only sensitive to carbapenems but also to other antibiotics, wherefore using carbapenem did not show additional benefit effect in the therapy outcome [18, 19]. The application of an initial non-carbapenem antibiotic with a broad spectrum should be a more appropriate choice [20, 21]. For patients with septic shock at high risk of multidrug-resistant organisms, SSC guidelines recommend empiric treatment with two antibiotics with Gram-negative coverage, rather than just one. Given the increased incidence of multidrug-resistant organisms in many parts of the world and the association between treatment delay and adverse outcomes, a combination of drugs is often required for initial treatment to ensure that treatment includes at least one drug that is effective against the causative organism [14]. Due to widespread misunderstanding of the ultra-broad spectrum efficacy of carbapenem, a single antibiotic is often used, resulting in the omission of Gram-negative coverage.

In the clinical application of carbapenem, we should pay attention to the following points: firstly, carbapenem is not always the strongest antibiotic choice. The strongest antibiotic choice should be the most sensitive antibiotic for the pathogenic bacteria of this infection, with the highest tissue concentration on the infection focus [21]. Secondly, the problem of carbapenem resistance is becoming increasingly prominent, including non-fermented bacteria [22, 23] as carbapenem-resistant *Klebsiella pneumoniae* and *Escherichia coli*. These increased rapidly in the hospitals, wherefore even in the treatment of non-fermented bacterial infections, carbapenem is not the best choice [24, 25]. Conversely, in the case of carbapenem resistance, it is the worst therapy option. Thirdly, extensive use of carbapenem without critical considerations and control, the situation of carbapenem resistance will deteriorate rapidly. Previous studies have already shown that the likelihood of developing carbapenem-resistant *Klebsiella pneumoniae* and *Escherichia coli* was significantly related to the used amount of carbapenem. Fourth, SSC guidelines recommend rapid identification of infection source requiring urgent control in patients with septic shock and the implementation of any necessary source control intervention as soon as medically technical and logistic feasible [14]. However, due to the widespread misunderstanding of the efficacy of carbapenems and the fluke psychology after the use of carbapenems, the source control intervention is often delayed.

Another explanation for using carbapenem as the preferred antibiotics did not show effect on the mortality of septic shock is probably associated with the availability of carbapenem in China. Early administration of appropriate antimicrobials is one of the most effective interventions to reduce mortality in patients with septic shock [26, 27]. For every additional hour from emergency department admission to antibiotics, in-hospital mortality increased onefold [28]. SSC guidelines recommend antimicrobials given within 1 h to patients with septic shock [14]. Due to the various difficulties of obtaining carbapenem, non-carbapenem antibiotics can often be used in clinical practice in a shorter period of time. Therefore, in the treatment of septic shock, using carbapenem antibiotics without evidence should be strictly prohibited to avoid resistance and abuse on the one hand. And on the other hand, the availability of carbapenem antibiotics should be improved for those patients with evidence for use, to achieve ultimately the purpose of availability without abuse.

The 2009 Extended Prevalence of Infection in Intensive Care study identified Gram-negative bacterial infections (e.g., *Escherichia coli*, *Enterobacter* spp., *Klebsiella* spp., *Pseudomonas aeruginosa*, and *Acinetobacter baumannii*) as the most common cause of sepsis with a frequency of 62%, followed by Gram-positive infections (mainly *Staphylococcus aureus*). Gram-negative bacilli are further divided into non-fermentative bacteria and fermentative bacteria. In our present study, 131 of the 782 hospitals (for which data were available) were dominated by non-fermentative bacteria, which is a group of Gram-negative bacteria does not ferment sugar. Non-fermentative Gram-negative bacilli are one of the leading causes of hospital-acquired infections [29–31]. There are four major bacteria belonging to this group: *Acinetobacter baumannii*, *Pseudomonas aeruginosa*, *Stenotrophomonas maltophilia*, and *Burkholderia cepacia*. They exist widely in nature and are important pathogens causing hospital infections. With the development of treatment technology and the wide application of new antimicrobial agents, incidence and drug resistance of non-fermentative bacterial infections have increased significantly in recent years [29, 30, 32]. Due to the generally existing multi-drug and even pan-drug resistance of non-fermentative bacteria, their increasing incidence has become a serious problem in the field of anti-infective therapy [33–35]. In our study, non-fermentative bacterial infection increased the mortality of septic shock, which was consistent with relevant previous study findings [30, 34]. Further research on septic shock caused by non-fermentative bacteria should be carried out in the future in order to explore how to achieve better therapy efficacy.

There are several limitations to this study. First, since the data of the time period of only one year were presented in this study, the relationships between antibiotics, pathogenic bacteria and mortality of septic shock could not be analyzed from a dynamic perspective. Second, this was an observational study and, therefore, prone to selection bias.

## Conclusion

In conclusion, using carbapenem as the preferred antibiotic in the treatment of septic shock did not show effect on the patient mortality of septic shock. Compared with patients with fermenting bacteria as the most common pathogenic bacteria, patients of septic shock with non-fermenting bacteria had a higher mortality.

## Data Availability

The datasets supporting the conclusions of this article are included within the article and its additional files.

## Abbreviations

ESICM: European Society for Intensive Critical Care
China-NCCQC: China National Critical Care Quality Control Center
QICC: Quality Improvement of Critical Care Program
ICU: Intensive care unit
APACHE: Program, Acute Physiology and Chronic Health Evaluation
